# A 6-Nucleotide Regulatory Motif within the AbcR Small RNAs of *Brucella abortus* Mediates Host-Pathogen Interactions

**DOI:** 10.1128/mBio.00473-17

**Published:** 2017-06-06

**Authors:** Lauren M. Sheehan, Clayton C. Caswell

**Affiliations:** Department of Biomedical Sciences and Pathobiology, Center for Molecular Medicine and Infectious Diseases, VA-MD College of Veterinary Medicine, Virginia Tech, Blacksburg, Virginia, USA; Washington State University; Ohio State University

**Keywords:** AbcR1, AbcR2, *Brucella*, *Alphaproteobacteria*, sRNA

## Abstract

In *Brucella abortus*, two small RNAs (sRNAs), AbcR1 and AbcR2, are responsible for regulating transcripts encoding ABC-type transport systems. AbcR1 and AbcR2 are required for *Brucella* virulence, as a double chromosomal deletion of both sRNAs results in attenuation in mice. Although these sRNAs are responsible for targeting transcripts for degradation, the mechanism utilized by the AbcR sRNAs to regulate mRNA in *Brucella* has not been described. Here, two motifs (M1 and M2) were identified in AbcR1 and AbcR2, and complementary motif sequences were defined in AbcR-regulated transcripts. Site-directed mutagenesis of M1 or M2 or of both M1 and M2 in the sRNAs revealed transcripts to be targeted by one or both motifs. Electrophoretic mobility shift assays revealed direct, concentration-dependent binding of both AbcR sRNAs to a target mRNA sequence. These experiments genetically and biochemically characterized two indispensable motifs within the AbcR sRNAs that bind to and regulate transcripts. Additionally, cellular and animal models of infection demonstrated that only M2 in the AbcR sRNAs is required for *Brucella* virulence. Furthermore, one of the M2-regulated targets, BAB2_0612, was found to be critical for the virulence of *B. abortus* in a mouse model of infection. Although these sRNAs are highly conserved among *Alphaproteobacteria*, the present report displays how gene regulation mediated by the AbcR sRNAs has diverged to meet the intricate regulatory requirements of each particular organism and its unique biological niche.

## IMPORTANCE

Small RNAs (sRNAs) are important components of bacterial regulation, allowing organisms to quickly adapt to changes in their environments. The AbcR sRNAs are highly conserved throughout the *Alphaproteobacteria* and negatively regulate myriad transcripts, many encoding ABC-type transport systems. In *Brucella abortus*, AbcR1 and AbcR2 are functionally redundant, as only a double *abcR1 abcR2* (*abcR1*/*2*) deletion results in attenuation *in vitro* and *in vivo*. In the present study, we confirmed that the AbcR sRNAs have redundant regulatory functions and defined two six-nucleotide motifs, M1 and M2, that the AbcR sRNAs utilize to control gene expression. Importantly, only M2 was linked to *B. abortus* virulence. Further investigation of M2-regulated targets identified BAB2_0612 as critical for colonization of *B. abortus* in mice, highlighting the significance of AbcR M2-regulated transcripts for *Brucella* infection. Overall, our findings define the molecular mechanism of the virulence-associated AbcR system in the pathogenic bacterium *B. abortus*.

## INTRODUCTION

Regulatory small RNAs (sRNAs) are important components of bacterial gene regulation, allowing organisms to quickly shift gene expression in response to changes in environmental conditions. sRNAs are typically less than 500 nucleotides in length and are capable of posttranscriptionally regulating mRNA targets by complementary, imperfect base pairing (1, 2), and these sRNA-mRNA interactions are commonly facilitated by the RNA chaperone Hfq (3–5). Once bound, mRNAs can experience one or more of several fates: sRNA can relieve hairpin structures in the untranslated region (UTR) of transcripts, allowing the ribosomal binding site (RBS) to be relaxed and translation to occur; sRNAs can block the RBS and inhibit binding of the ribosome, thus impeding the start of translation; and/or sRNAs can bind to either the UTR or coding region (CDR) of the transcript and target the mRNA or the entire sRNA-mRNA complex for degradation by an RNase (6–8).

AbcR1 and AbcR2 are two highly conserved sRNAs found throughout species in the class of *Alphaproteobacteria* (9–11). Although the AbcR sRNAs are similar in nucleotide composition and secondary structure among bacteria, they have diverged greatly in their regulatory capacity. In the plant pathogen *Agrobacterium tumefaciens*, AbcR1, but not AbcR2, has been shown to regulate gene expression (11). *A. tumefaciens* AbcR1 regulates expression of ABC-type transporter genes, the majority of which encode amino acid and sugar uptake systems. In contrast, AbcR1 and AbcR2 in the plant symbiont *Sinorhizobium meliloti* are both responsible for regulating specific and shared transcripts (12, 13).

In plant-associated *Alphaproteobacteria* such as *A. tumefaciens* and *S. meliloti*, AbcR1 and AbcR2 are chromosomally encoded in tandem, directly downstream of a gene encoding a LysR-type transcriptional regulator. In the human pathogen *Brucella abortus*, *abcR1* is located on chromosome II, while *abcR2* is positioned on chromosome I. In *B. abortus*, expression of *abcR2*, but not *abcR1*, is controlled by VtlR, the orthologous LysR-type transcriptional regulator found upstream of the genes encoding the *A. tumefaciens* and *S. meliloti* AbcR sRNAs (14). These differences in regulatory roles, genetic organization, and transcriptional regulation highlight the evolutionary differentiation of the AbcR regulatory system, which may have helped drive the host-bacterium relationships of *Alphaproteobacteria*.

In *B. abortus*, AbcR1 and AbcR2 negatively regulate several mRNA transcripts and are critical for the ability of the bacteria to cause a chronic infection in mice (15). Although *abcR1* and *abcR2* are located on different chromosomes, the sRNAs exhibit a high degree of nucleotide identify and very similar secondary structures, suggesting regulatory redundancy. Importantly, these two sRNAs seem to be functionally redundant, as isogenic chromosomal mutations in *abcR1* or *abcR2* do not affect the virulence of *B. abortus* but a double deletion of both *abcR1* and *abcR2* (Δ*abcR1*/*2*) results in attenuation *in vitro* and *in vivo*. Microarray analysis of *B. abortus* Δ*abcR1*/*2* revealed over 20 transcripts with elevated levels, demonstrating negative regulation by the sRNAs. Importantly, this regulation is posttranscriptional, as the results from the microarray analyses closely mirrored the results of the quantitative proteomic analyses.

For this study, we chose to focus on four AbcR targets that were identified as being upregulated over 3-fold in both microarray analysis and quantitative proteomics: BAB1_0314, BAB1_1794, BAB2_0612, and BAB2_0879. Importantly, although over 20 transcripts were found to be differently expressed in the *B. abortus abcR1*/*2* deletion strain, these four targets appear to be part of four independent AbcR-regulated systems. *bab1_0314* is predicted to encode subunit A of a dihydropyrimidine dehydrogenase along with *bab1_0313*, *bab1_1794* is predicted to encode a periplasmic binding protein of a putative ABC-type transport system encoded by *bab1_1794* to *bab1_1799*, BAB2_0612 is a putative glutamate-binding protein predicted to be a part of an ABC-type transport system, and BAB2_0879 is a putative polyamine-binding protein predicted to be a part of an ABC-type transport system encoded by *bab2_0874* to *bab2_0879*. Although AbcR1 and AbcR2 are known to play a critical role in promoting degradation of these transcripts in *B. abortus*, the regulatory redundancy and the mechanism by which the sRNAs target mRNAs for degradation have not been characterized.

As discussed above, AbcR1 and AbcR2 are important regulatory components in *B. abortus* and are critical for the ability of the brucellae to cause infection. Although the targets of AbcR-mediated gene regulation have been defined, the mechanism of regulatory redundancy remains uncharacterized. Here, we determined that AbcR1 and AbcR2 exhibit redundant mechanisms to control gene expression, and we have defined two nucleotide sequence motifs, called M1 and M2, that are required for AbcR-mRNA interactions. Most importantly, M2, but not M1, is critical for the wild-type virulence of *Brucella abortus*. To date, this is the first demonstration of a molecular mechanism of sRNA-mediated gene expression in *Brucella*.

## RESULTS

### AbcR1 and AbcR2 share redundant regulatory functions in *B. abortus* 2308.

**FIG 1:**
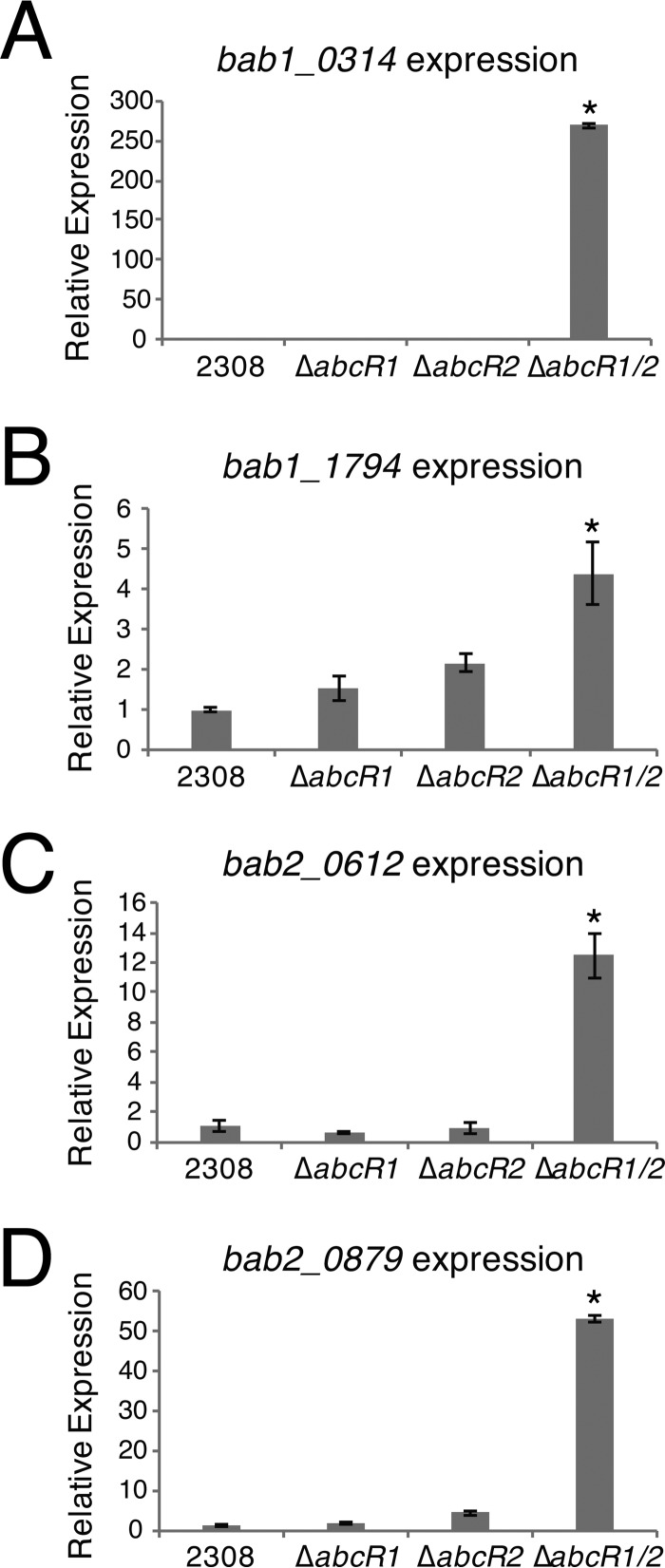
The AbcR sRNAs serve redundant regulatory functions in *Brucella abortus*. *Brucella abortus* 2308, Δ*abcR1*, Δ*abcR2*, and Δ*abcR1*/*2* strains were grown in brucella broth to an appropriate phase of growth, and RNA was extracted and reverse transcribed to cDNA. Quantitative reverse transcriptase PCR (qRT-PCR) was carried out with gene-specific primers for amplification of (A) *bab1_0314*, (B) *bab1_1794*, (C) *bab2_0612*, and (D) *bab2_0879*. Amplification was achieved utilizing incorporation of SYBR green. Relative expression levels of the AbcR targets were normalized to that of *Brucella abortus* 2308, and 16S rRNA was used as a control. Data represent the average relative levels of expression from each *B. abortus* strain ± the standard deviations of results from triplicate samples. Statistical significance was evaluated using one-way analysis of variance (ANOVA) followed by the Tukey-Kramer posttest. Asterisks denote significance (*P* < 0.0001).

Caswell et al. demonstrated that chromosomal isogenic deletion of either *abcR1* or *abcR2* in *B. abortus* resulted in a wild-type virulence phenotype but that a double *abcR1 abcR2* mutant led to significant attenuation in a mouse model of infection (15). Although this suggested functional redundancy, it was unknown if one or both of the sRNAs regulate the identified mRNA targets, which were identified using the *abcR1 abcR2* double mutant strain. To determine this, RNA from *B. abortus* 2308, Δ*abcR1*, Δ*abcR2*, and Δ*abcR1*/*2* was reverse transcribed to cDNA, and quantitative reverse transcriptase PCR (qRT-PCR) was performed on the four most highly regulated transcripts: *B. abortus b1_0314* (*bab1_0314*), encoding a putative oxidoreductase; *bab1_1794*, encoding a putative amino acid-binding protein; *bab2_0612*, encoding a putative glutamate-binding protein; and *bab2_0879*, encoding a putative polyamine-binding protein (Fig. 1). Individual deletions of either *abcR1* or *abcR2* did not lead to altered target mRNA levels; however, a strain in which both *abcR1* and *abcR2* were deleted exhibited statistically significantly increased levels of the mRNA targets compared to wild-type strain 2308, clearly demonstrating that AbcR1 and AbcR2 possess redundant regulatory functions in *B. abortus*.

### AbcR1 and AbcR2 directly bind to mRNA targets.

**FIG 2:**
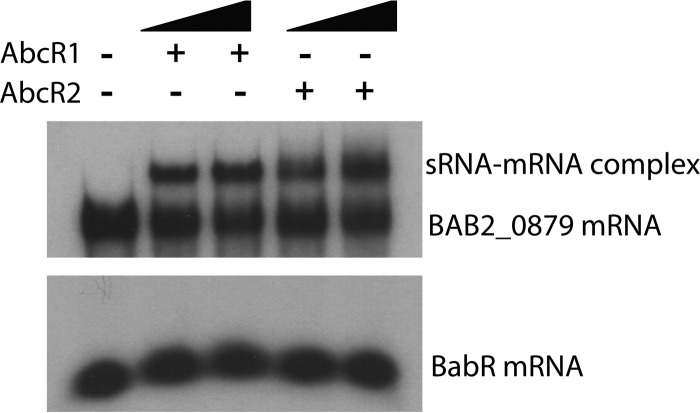
AbcR1 and AbcR2 bind to BAB2_0879 mRNA in a concentration-dependent manner. (A) *In vitro* transcribed AbcR1 and AbcR2 were assessed for their ability to individually bind radiolabeled BAB2_0879 mRNA (~1 ng). Increasing amounts (500-fold to 1,000-fold excess) of either AbcR1 or AbcR2 were added to BAB2_0879 mRNA, and the reaction mixture was heated to 90°C for 2 min and then incubated at room temperature for 30 min. (B) Radiolabeled BabR mRNA (~1 ng) was used as a control.

Electrophoretic mobility shift assays (EMSAs) were employed to assess the ability of the *B. abortus* AbcR sRNAs to directly bind to target mRNAs, specifically, to the 5′ UTR of the BAB2_0879 mRNA (Fig. 2). BAB2_0879 RNA was radiolabeled using *in vitro* transcription that employed the T7 promoter, and the labeled BAB2_0879 RNA was incubated with increasing concentrations of *in vitro* transcribed AbcR1 or AbcR2. Both AbcR1 and AbcR2 exhibited concentration-dependent binding to BAB2_0879 mRNA. To ensure that sRNA-mRNA binding was specific to the AbcR-regulated target RNA, a negative-control binding reaction was performed with RNA corresponding to the 5′ UTR of BabR, a *Brucella* LuxR-type transcriptional regulatory protein. No binding between the AbcR sRNAs and BabR RNA was observed in this experiment (Fig. 2, lower panel). Overall, these data demonstrate the direct interaction of the AbcR sRNAs with a target mRNA in *B. abortus*.

### Identification of two regulatory sequences in the AbcR sRNAs.

**FIG 3:**
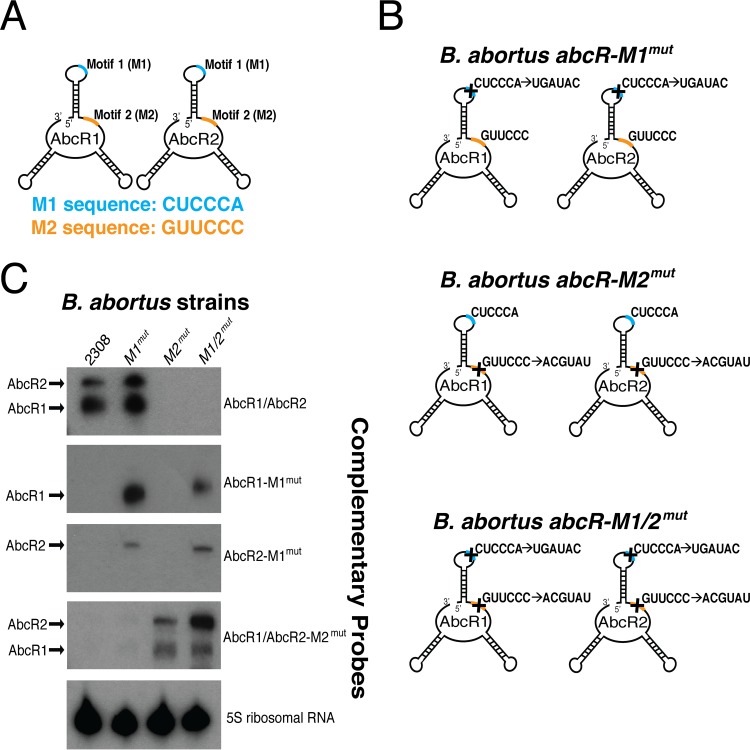
The AbcR sRNAs contain two putative 6-nucleotide binding motifs. (A) Schematic of AbcR sRNAs encoding two conserved motifs: motif 1 (M1: CUCCCA) (shown in cyan) and motif 2 (M2: GUUCCC) (shown in orange). M1 is a 6-nucleotide single-strand RNA sequence, located in the first hairpin on the sRNAs. M2 is also a 6-nucleotide single-strand RNA sequence, located between the first and second hairpins. (B) To determine the regulatory capacity of M1 and M2 in the AbcR sRNAs, site-directed mutagenesis was performed. All *B. abortus* strain names are listed above a schematic of the AbcR mutagenesis strategy. A "+" denotes the location where mutagenesis was performed. (C) Northern blot analysis was employed to ensure that mutagenesis of AbcR sRNAs did not result in degradation. RNA from *B. abortus* 2308, *abcR-M1*^*mut*^, *abcR-M2*^*mut*^, and *abcR-M1*/*2*^*mut*^ was separated on a denaturing polyacrylamide gel and transferred to a nitrocellulose membrane. Here, the specific motif mutations were probed for. The wild-type AbcR1/AbcR2 probe was specific to AbcR1 and AbcR2 in *B. abortus* 2308 (first panel). It should be noted that this probe sequence contains wild-type M2 (i.e., GUUCCC) but not wild-type M1. Thus, *abcR-M1*^*mut*^ was detected. A schematic of probe annealing locations can be found in Fig. S1.

In *A. tumefaciens*, two putative regulatory motifs were computationally predicted in AbcR1, and EMSAs revealed that AbcR1 employs both motifs to bind the 5′ UTRs or CDRs in mRNA transcripts (16). Analysis of the *B. abortus* AbcR sRNA secondary structures identified two conserved sequences comprised of 6 nucleotides each in both AbcR1 and AbcR2 (Fig. 3A). The first motif (M1), CUCCCA, is located in the first hairpin of the sRNAs, while the second motif (M2), GUUCCC, is found between the first and second hairpins. AbcR-regulated mRNA transcripts were subsequently examined to identify putative complementary binding sequences with respect to the AbcR sRNAs. Several transcripts were found to contain complementary M1 and/or M2 sequences in either their 5′ UTR or within the CDR (see Table S1 in the supplemental material). This variability in the location of binding sites is not uncommon for negatively regulated transcripts, as sRNAs can bind and block ribosome at the Shine-Dalgarno sequence in the 5′ UTR or can cause early termination of translation and targeted degradation by binding to the CDR of the mRNA (6–8).

10.1128/mBio.00473-17.2 TABLE S1  Putative motifs in AbcR-regulated transcripts. All complementary motifs are bold and underlined. Download TABLE S1, PDF file, 0.2 MB. Copyright © 2017 Sheehan and Caswell. 2017 Sheehan and Caswell This content is distributed under the terms of the Creative Commons Attribution 4.0 International license.

10.1128/mBio.00473-17.1 FIG S1  Annealing location of Northern blot probes in *B. abortus* strains. Nucleotide sequences of AbcR1 and AbcR2 are listed for each *B. abortus* strain (left). The nucleotides for motif 1 (M1) are bold and highlighted in cyan, while the nucleotides for motif 2 (M2) are bold and highlighted in orange. The black bolded nucleotides represent site-directed mutagenesis of either M1 (CUCCCA to UGAUAC) or M2 (GUUCCC to ACGUAU). A red bar represents the Northern blot probe and is located where annealing of the probe to the AbcR sRNA occurs. If no red bar is present, this indicates no annealing of the probe to the sRNA due to inadequate complementary binding. Download FIG S1, PDF file, 1.1 MB. Copyright © 2017 Sheehan and Caswell. 2017 Sheehan and Caswell This content is distributed under the terms of the Creative Commons Attribution 4.0 International license.

### The AbcR sRNAs regulate expression of mRNAs using the M1 and M2 motifs.

Following identification of the M1 and M2 motifs in the AbcR sRNAs, we sought to determine the involvement, if any, of the motifs in AbcR-mediated gene regulation (Fig. 3). Site-directed mutagenesis was carried out on both AbcR sRNAs in *B. abortus* to mutate the M1 CUCCCA sequence to a nonsense sequence, UGAUAC (denoted as *abcR*-*M1*^*mut*^). Likewise, the M2 GUUCCC sequence was mutated to ACGTAT (denoted as *abcR*-*M2*^*mut*^). Finally, a double-motif mutant was constructed (denoted as *abcR-M1*/*2*^*mut*^) (Fig. 3B). Northern blot analyses were performed on all AbcR motif mutant strains to ensure that sequence changes did not negatively affect sRNA transcription and stability (Fig. 3C). Indeed, all *B. abortus* strains produced the corresponding AbcR sRNA.

**FIG 4:**
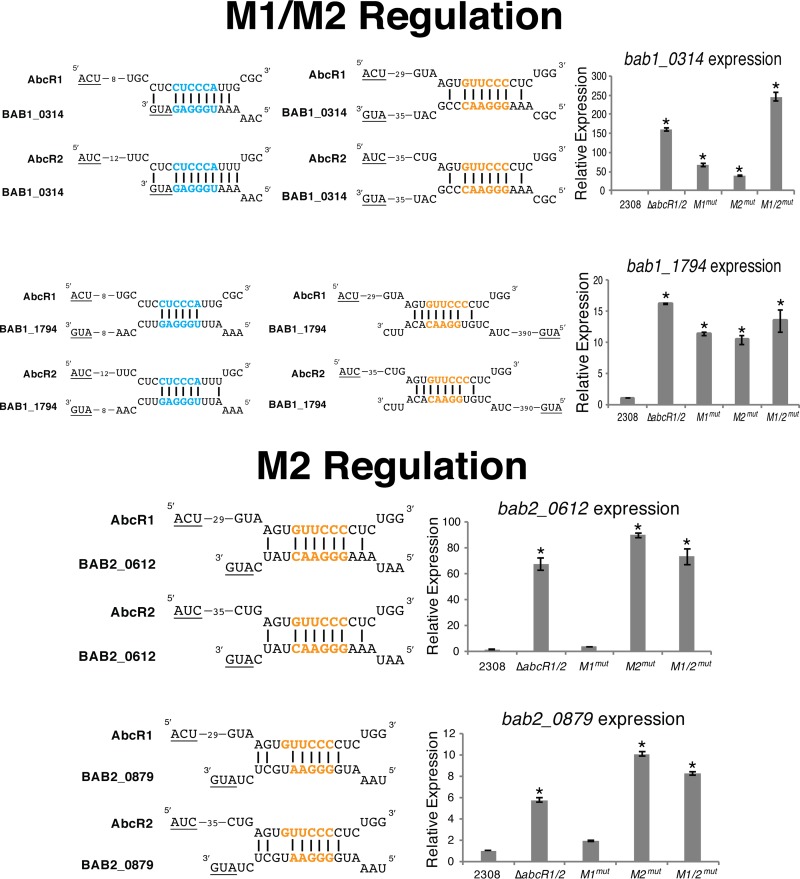
The AbcR sRNAs utilize M1 and/or M2 to regulate mRNA targets. To evaluate M1 and M2 in the regulation of AbcR-specific mRNA targets, qRT-PCR was employed on *B. abortus* 2308, Δ*abcR1*/*2*, *abcR-M1*^*mut*^, *abcR-M2*^*mut*^, and *abcR-M1*/*2*^mut^ for amplification of *bab1_0314*, *bab1_1794*, *bab2_0612*, and *bab2_0879*. Underlined nucleotides denote the start codon. M1 motifs are denoted by cyan nucleotides; M2 motifs are denoted by orange nucleotides. Relative expression levels of all genes were normalized to that of *B. abortus* 2308, and 16S rRNA was used as a control. Data represent the average relative levels of expression from each *B. abortus* strain ± the standard deviations of results from triplicate samples. Statistical significance was evaluated using one-way analysis of variance (ANOVA) followed by the Tukey-Kramer posttest. Asterisks denote significance (*P* < 0.0001).

Following sRNA mutagenesis, qRT-PCR was employed to determine if gene expression of the mRNA targets was affected by changes made to the M1 and M2 putative binding motifs (Fig. 4; Table 1). These data showed that the AbcR sRNAs regulated transcripts by utilizing M1 or M2 or a combination of M1 and M2. Expression of *bab1_1799*, encoding a putative permease of an amino acid ABC transport system, was the sole mRNA target found to be regulated by M1 (Table 1). Expression levels of *bab2_0506*, *bab2_0612*, *bab2_0879*, and *bab2_1062* were significantly upregulated in the *abcR-M2*^*mut*^ strain compared to wild-type strain 2308, suggesting that these mRNA targets are regulated solely by M2. Finally, expression levels of *bab1_0313*, *bab1_0314*, *bab1_1794*, and *bab2_0491* were significantly upregulated in both the *abcR-M1*^*mut*^ and *abcR-M2*^*mut*^ strains, suggesting that both motifs are utilized by the AbcR sRNAs to regulate expression of these targets.

**TABLE 1:** qRT-PCR for additional targets regulated by the AbcR sRNAs^a^

Gene	Relative expression level of *Brucella abortus* strain:				
	2308	Δ*abcR1*/*2*	*M1^mut^*	*M2^mut^*	*M1*/*2*^*mut*^
*bab1_0313*	1.00	143.69	39.72	13.05	181.01
*bab1_1799*	1.00	1.74	2.29	0.76	2.47
*bab2_0491*	1.00	3.47	2.69	4.13	3.69
*bab2_0506*	1.00	2.16	0.99	2.56	2.12
*bab2_1062*	1.00	3.53	0.67	4.32	3.98
16S rRNA	1.00	0.99	0.98	0.98	0.99

a qRT-PCR was carried out with *B. abortus* 2308, Δ*abcR1*/*2*, *abcR-M1*^*mut*^, *abcR-M2*^*mut*^, *abcR-M1*/*2*^mut^ to determine expression of the remaining mRNA targets previously identified by microarray analysis. The expression levels of all genes were normalized to that of *B. abortus* 2308, and 16S rRNA was used as a control.

**FIG 5:**
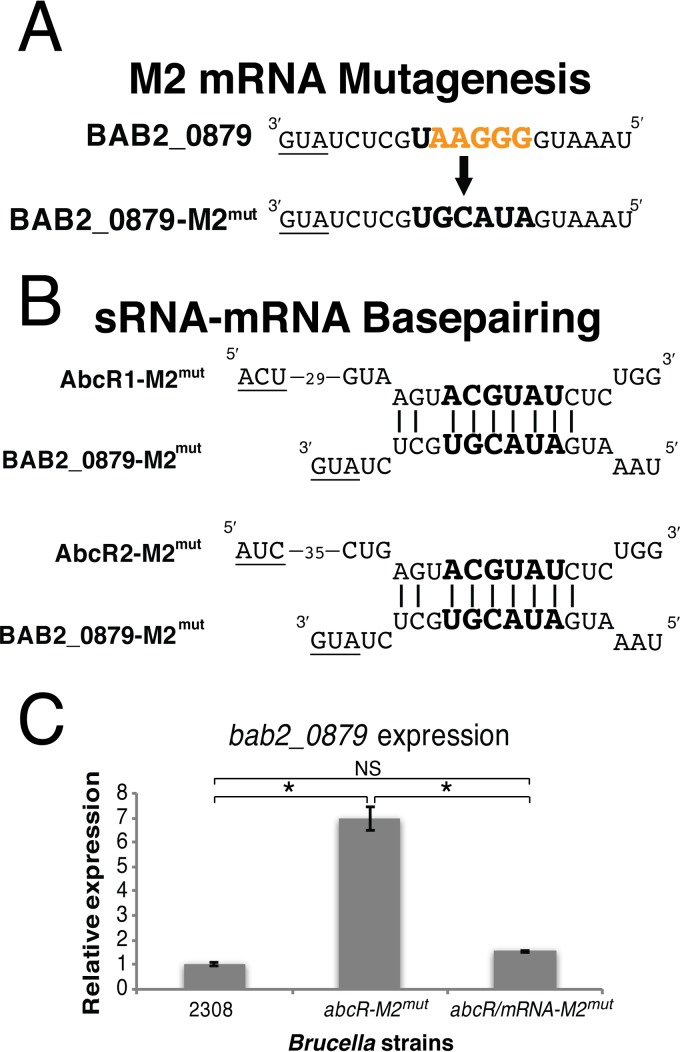
Mutagenesis of M2 in *bab2_0879* results in reestablishment of negative regulation in the *B. abortus abcR-M2*^*mut*^ strain. (A) Illustration of site-directed mutagenesis carried out on the putative M2 site in *bab2_0879*. For this, the putative M2 site, located in the 5′ UTR of *bab2_0879*, was mutated from UAAGGG to UGCAUA. (B) This mutated sequence, which is complementary to M2 in the *abcR-M2*^*mut*^ strain, was predicted to result in the reestablishment of regulation by the AbcR sRNAs. (C) qRT-PCR was employed on *B. abortus* 2308, *abcR-M2*^*mut*^, and *abcR*/*bab2_0879-M2*^*mut*^ for amplification of *bab2_0879*. Relative expression levels were normalized to that of *B. abortus* 2308, and 16S rRNA was used as a control. Data represent the average relative levels of expression from each *B. abortus* strain ± the standard deviations of results from triplicate samples. Statistical significance was evaluated using one-way analysis of variance (ANOVA) followed by the Tukey-Kramer posttest. Asterisks denote significance (*P* < 0.0001). NS, not significant.

To further validate these findings, a *B. abortus* double M2 motif mutant of the AbcR sRNAs and BAB2_0879 mRNA was constructed to assess if negative regulation could be restored (Fig. 5). For mRNA mutagenesis, the wild-type *B. abortus* BAB2_0879 M2 motif, UAAGGG, was mutated to UGCAUA (Fig. 5A). The BAB2_0879 motif was mutated to be complementary to the M2 motif in the *B. abortus abcR-M2*^*mut*^ strain, in the hope that negative regulation by the AbcR sRNAs could be reestablished (Fig. 5B). Following mutagenesis, qRT-PCR was performed to analyze expression of *bab2_0879* in the *B. abortus* double *abcR mRNA-M2* mutant. As expected, downregulation of BAB2_0879 was restored. Taken together, these experiments identified and confirmed two 6-nucleotide motifs, M1 and M2, within the AbcR sRNAs that are required for regulation of mRNA targets in *B. abortus*.

### Deletion of M2, but not M1, in the AbcR sRNAs results in attenuation of *Brucella abortus* in mice.

**FIG 6:**
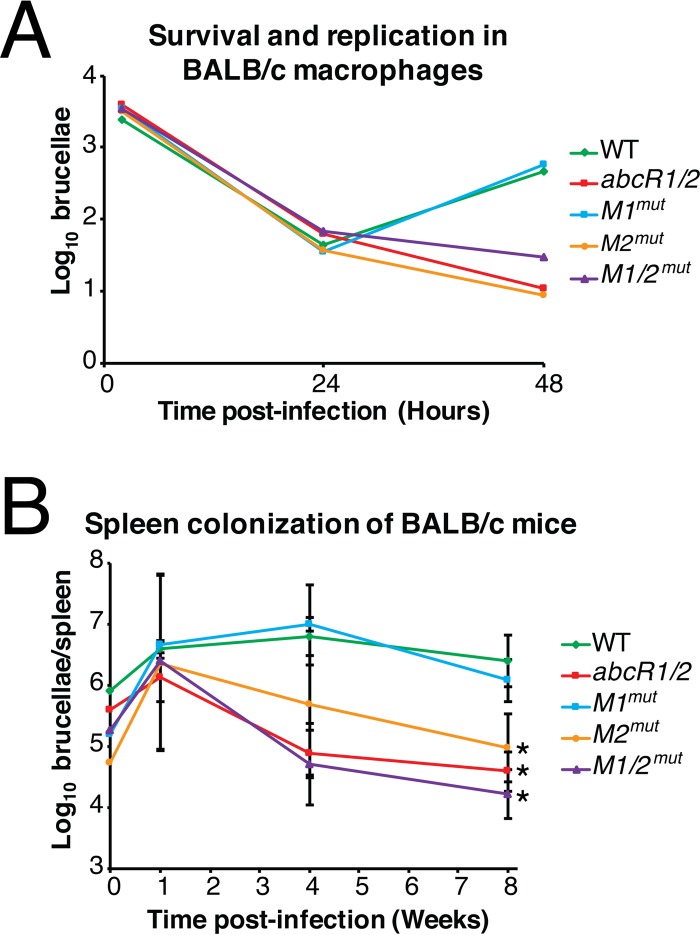
M2, but not M1, in the AbcR sRNAs is involved in *Brucella* pathogenesis. (A) Naive, peritoneal BALB/c macrophages were infected with *B. abortus* 2308, Δ*abcR1*/*2*, *abcR-M1*^*mut*^, *abcR-M2*^*mut*^, or *abcR-M1*/*2*^mut^ to evaluate survival and colonization of the brucellae. At 2, 24, and 48 h postinfection, macrophages were lysed with deoxycholate, and serial dilutions were made on SBA to determine the number of intracellular brucellae. WT, wild type. (B) Spleen colonization of BALB/c mice by *Brucella abortus abcR* motif mutants. Six-week-old BALB/c mice were infected with *B. abortus* strains via intraperitoneal infections. At 1, 4, and 8 week postinfection, mice were sacrificed and spleen homogenates were serially diluted to determine the number of brucellae per spleen. Data represent average CFU counts per spleen ± the standard deviations of results from five mice infected with each *Brucella* strain. Statistical significance was evaluated using one-way analysis of variance (ANOVA) followed by the Tukey-Kramer posttest. Asterisks denote significance (*P* < 0.05).

As demonstrated previously, AbcR1 and AbcR2 are essential for the wild-type virulence of *Brucella abortus* (15). The potential reason for the attenuation seen with a *B. abortus abcR1 abcR2* double deletion may be the upregulation of a numerous and rather diverse set of ABC-type transport systems. Since the M1 and M2 motifs in the sRNAs were demonstrated to be involved in regulation of transcripts (Fig. 4), it was hypothesized that mutagenesis of the M1 and M2 motifs in the AbcR sRNAs would affect the virulence of *B. abortus* (Fig. 6). To test this hypothesis, BALB/c mouse macrophages were infected with the AbcR sRNA motif mutants (Fig. 6A). Surprisingly, while the *B. abortus abcR-M1*^*mut*^ trended with the wild-type strain, the *B. abortus abcR-M2*^*mut*^ strain was unable to successfully infect and survive in macrophages. Furthermore, similar results were observed in a mouse model of infection, where the *abcR-M2*^*mut*^ strain exhibited a decreased ability to chronically infect mice (Fig. 6B). These data suggest that the 6-nucleotide M2 motif in AbcR sRNAs is required for the full virulence of *B. abortus*.

### An AbcR M2-regulated target, BAB2_0612, is critical for colonization of *Brucella abortus* in mice.

**FIG 7:**
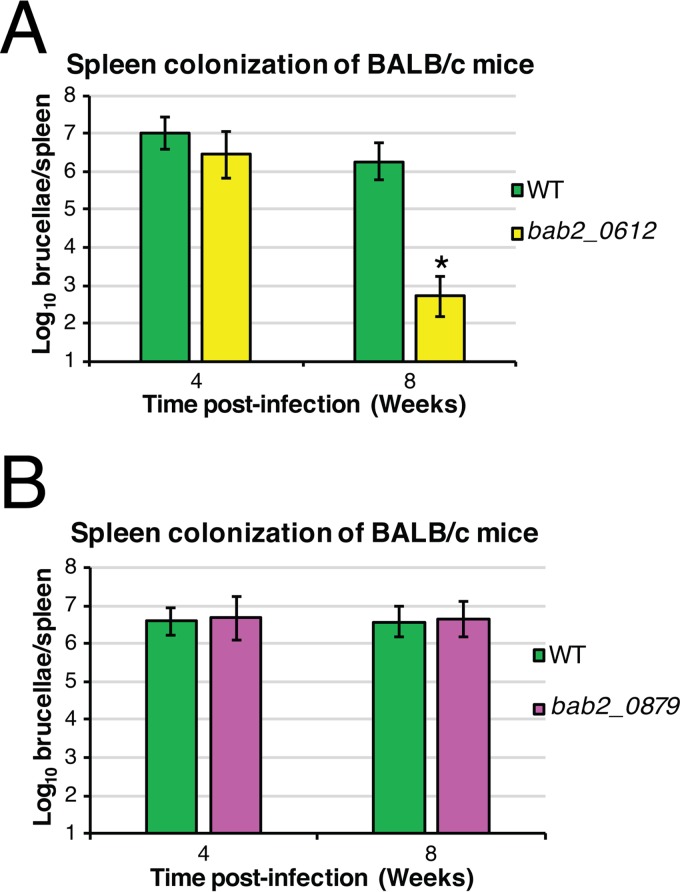
*Brucella abortus* Δ*bab2_0612* is unable to cause a wild-type chronic infection in BALB/c mice. (A) Spleen colonization of BALB/c mice by *Brucella abortus* Δ*bab2_0612*. Six-week-old BALB/c mice were infected with *B. abortus* strains via intraperitoneal infections. At 4 and 8 weeks postinfection, mice were sacrificed and spleen homogenates were serially diluted to determine the number of brucellae per spleen. Data represent average CFU counts per spleen ± the standard deviations of results from five mice infected with each *Brucella* strain for the 4-week time point and from four mice infected with each *Brucella* strain for the 8-week time point. Statistical significance was evaluated using one-way analysis of variance (ANOVA) followed by the Tukey-Kramer posttest. Asterisks denote significance (*P* < 0.05). (B) Spleen colonization of BALB/c mice by *Brucella abortus* Δ*bab2_0879*. Six-week-old BALB/c mice were infected with *B. abortus* strains via intraperitoneal infections. At 4 and 8 weeks postinfection, mice were sacrificed and spleen homogenates were serially diluted to determine the number of brucellae per spleen. Data represent average CFU counts per spleen ± the standard deviations of results from five mice infected with each *Brucella* strain.

Following the demonstration that M2 in the AbcR sRNAs is critical for *Brucella* pathogenesis, we sought to determine if deletion of any of the M2-regulated targets would result in attenuation in a mouse model of infection (Fig. 7). Isogenic, unmarked, in-frame gene deletions were made of two M2-regulated targets: *bab2_0612*, encoding a putative glutamate-binding protein, and *bab2_0879*, encoding a putative polyamine-binding protein. Subsequently, the strains were assessed for their ability to cause a chronic infection in mice. *B. abortus* Δ*bab2_0612* was significantly attenuated in BALB/c mice compared to wild-type strain 2308 (Fig. 7A), whereas *B. abortus* Δ*bab2_0879* showed no difference from the wild-type strain in colonization (Fig. 7B). These data suggest that BAB2_0612 is a critical component of *Brucella* virulence.

## DISCUSSION

The AbcR small RNAs were previously shown to be vital components of the virulence of *B. abortus* and, moreover, to be involved in negatively regulating transcripts largely encoding ABC-type transport systems (15). Importantly, and as further defined in this study, the AbcR sRNAs are a prime example of sibling sRNAs, as they share identical regulatory repertoires in *B. abortus* 2308 (15, 17) (Fig. 1). While the link between the AbcR sRNAs and *Brucella* pathogenesis is evident, it was unknown how the AbcR sRNAs target and bind transcripts, ultimately leading to mRNA degradation.

In the present study, we identified and experimentally confirmed that two motifs, M1 and M2, which are comprised of six nucleotides each, are the primary sequences used by the AbcR sRNAs to bind transcripts in *B. abortus* (Fig. 3 to 5; Table 1). In the plant pathogen *A. tumefaciens*, AbcR1 is the main regulatory AbcR sRNA, as AbcR1 regulates transcripts encoding a diverse set of nutrient uptake systems (11, 16). In contrast, AbcR2 has not been reported to have any regulatory function in *A. tumefaciens*. Although AbcR2 is speculated to have arisen from gene duplication, it is important to note a major difference in the *A. tumefaciens* AbcR2 that may explain its lack of regulatory capability: the M1 sequence is absent. In contrast, both M1 and M2 are present in *A. tumefaciens* AbcR1. Importantly, the M1 and M2 sequences in *A. tumefaciens* AbcR1 are identical to the M1 and M2 sequences in the *B. abortus* AbcR sRNAs. However, *A. tumefaciens* AbcR2 contains only the M2 site. This absence of M1 may contribute to the lack of a regulatory function by AbcR2 in *A. tumefaciens*. Furthermore, because of the importance that the M2 site has in *Brucella* virulence (Fig. 6), it will be interesting to learn what role, if any, the AbcR M2 motif plays in *Agrobacterium* biology and pathogenesis.

In the plant symbiont *S. meliloti*, AbcR1 has been hypothesized to utilize M1 to bind transcripts (12). However, there is currently no experimental evidence to confirm this interaction. As is the case in *A. tumefaciens*, AbcR1 is defined as having the dominant regulatory role in *S. meliloti*. However, AbcR2 was also shown to have some regulatory capacity as well, although not to the same degree as AbcR1. This difference in AbcR regulation levels may also be linked to the two motifs. M1 and M2 are present in *S. meliloti* AbcR1 and are identical to the AbcR motifs in both *Agrobacterium* and *Brucella*. Conversely, the M1 sequence in AbcR2 is mutated from CUCCCA to CUCCCC. Though this difference is in one nucleotide, it may compromise the regulatory ability of AbcR2, but this remains to be experimentally determined.

In *B. abortus*, the AbcR sRNAs are interesting examples of sibling sRNAs (17), as the two *Brucella* AbcR sRNAs have identical regulatory roles and utilize two conserved motifs for binding transcripts. Although the regulatory profiles of M1 and M2 are not identical (Fig. 4; Table 1), only M2 was found to be critical for the virulence of *B. abortus* in both macrophages and mice (Fig. 6). The attenuation seen in an *abcR-M2* mutant may be due to the overproduction of potentially immunogenic proteins such as BAB2_0506, BAB2_0612, BAB2_0879, and/or BAB2_1062. To start to investigate if any of these M2-regulated targets have a role in pathogenesis, isogenic gene deletions were made in two of the M2 targets: *bab2_0612* and *bab2_0879*. *bab2_0612* is predicted to encode a periplasmic binding protein of an ABC-type glutamate transport system. Aside from this prediction, nothing is currently known about BAB2_0612 in *Brucella*. Interestingly, in *A. tumefaciens*, Atu1879, the orthologue of BAB2_0612, is regulated by AbcR1, but the regulation is performed specifically by the M1 motif (16). However, the results of our present study show that BAB2_0612 in *Brucella* is controlled entirely by the M2 motif (Fig. 4). In mice, the Δ*bab2_0612* mutant is attenuated compared to wild-type strain (Fig. 7A), highlighting the importance of this M2-regulated target in infection. These seemingly small but strikingly important differences in M1-mediated versus M2-mediated gene regulation will need to be better defined in the *Alphaproteobacteria* in order to understand the evolutionary consequences of AbcR-linked gene expression for host specificity of the bacteria. In terms of *Brucella*, current work is aimed at further characterization of BAB2_0612, particularly by developing a *bab2_0612* overexpression strain and determining the function of this protein in *B. abortus*.

*bab2_0879* is predicted to encode a putative periplasmic protein in an ABC-type polyamine transport system. In bacteria, polyamines can be viewed a double-edged sword (18). Polyamines provide protection against free radicals (19, 20) and promote the expression of acid resistance genes during stress (21); both activities could be beneficial to intracellular organisms such as *Brucella*. The increase in the level of BAB2_0879 in the *abcR-M2* mutant could be linked to polyamine toxicity. However, this is strictly speculation as no studies have linked polyamine synthesis or transport to *Brucella* pathogenesis. Deletion of *bab2_0879* in *B. abortus* did not affect colonization of the bacteria in a mouse model of infection, but previous studies have shown that deletion of this gene in *B. melitensis* results in attenuation (22). However, because an *abcR-M2* mutant is hypothesized to overproduce BAB2_0879, deletion of these targets may not recapitulate what is happening in a *B. abortus abcR-M2* mutant strain. Current work is targeted at identifying and subsequently mutating complementary M2 motifs in all M2-regulated targets (i.e., BAB2_0506, BAB2_0612, BAB2_0879, and/or BAB2_1062) in *B. abortus* 2308 and at assessing the ability of these overexpressing *Brucella* strains to infect both macrophages and mice.

sRNAs typically regulate transcripts through the direct or indirect sensing of specific environmental stimuli (1, 2). It is by this mechanism that sRNAs can quickly alter the bacterial transcriptome to respond to and adapt to stress. In regard to the *Brucella* AbcR sRNAs, no specific stimuli have been identified that are responsible for altering their regulatory activities. Current research in our laboratory is aimed at identifying the stimuli responsible for changing the expression levels of the sRNAs. Importantly, we previously identified the transcriptional activator of *abcR2*, VtlR (14). VtlR belongs to the class of LysR-type transcriptional regulators, whose members typically have an N-terminal DNA-binding domain and a C-terminal coinducer binding domain. Current research is aimed at identifying the putative coinducer with which VtlR is interacting, which may in turn result in the alteration of expression of *abcR2*. Moreover, since external stimuli added to culture can never fully recapitulate intracellular environmental conditions, our laboratory is aiming to perform transcriptome sequencing (RNA-seq) from brucellae extracted from macrophages during various phases of infection. This experiment will give us insight into the transcriptome of *Brucella in vitro* and, more specifically, will give us a clear picture of the AbcR system during intracellular infection.

The AbcR sRNAs are involved in the negative regulation of mRNA transcripts, ultimately targeting them for degradation. It has been suggested that RNase E may be involved in AbcR sRNA degradation, as a *S. meliloti* Rnase E gene mutant was reported to show increased stability of AbcR1 (23). RNase E is a highly conserved bacterial endoribonuclease involved in the processing and decay of RNA (reviewed in reference 24). Moreover, pertaining to its association with sRNAs, the C terminus of RNase E has been reported to bind the sRNA chaperone Hfq and assist in sRNA-mRNA decay (25–28). Empirical evidence suggests that RNase E is essential in *B. abortus*, but, importantly, the data suggested that a nonlethal C-terminal truncation of RNase E could be constructed in *B. abortus* (Xavier DeBolle, personal communication). Therefore, to determine if RNase E (BAB1_0930) is responsible for AbcR sRNA-mRNA degradation in *B. abortus*, a chromosomal *B. abortus* Rnase E gene mutant was constructed which lacked the C-terminal domain. Northern blot analysis of the AbcR sRNAs, as well as qRT-PCR analysis for determination of mRNA levels of AbcR-regulated targets, found no association between RNase E and AbcR-mRNA degradation (data not shown). It is possible that the region associated with sRNA degradation was not deleted in this *B. abortus* strain, but we have not experimentally tested this hypothesis. In addition to RNase E, several other *B. abortus* RNases were also tested for their potential role in AbcR-mRNA degradation: YbeY, a putative single-strand and double-strand endoribonuclease (29–32); RNase J, a 5′–3′ exoribonuclease (reviewed in reference 33); and RNase R, a 3′–5′ hydrolytic exoribonuclease (reviewed in reference 34). None of those RNases were found to be associated with the AbcR sRNAs or with their targets (data not shown). Current work is focused on identifying the RNase involved in AbcR sRNA degradation in *B. abortus*.

**FIG 8:**
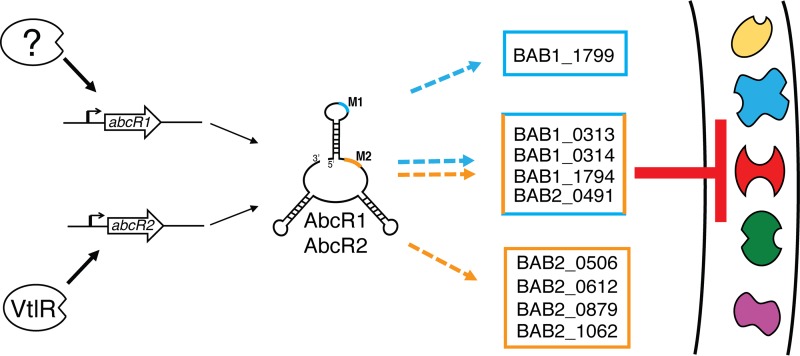
AbcR-mediated regulation in *Brucella abortus*. A working model illustrating AbcR sRNA regulation of mRNAs in *Brucella abortus* 2308 is shown. Following transcription of *abcR2* by VtlR and of *abcR1* by an unknown protein, the AbcR sRNAs utilize two conserved motifs, M1 (cyan) and M2 (orange), to bind and regulate target mRNAs, many of which encode components of ABC-type transport systems.

In summary, this work experimentally confirmed the presence of two regulatory motifs, comprised of only 6 nucleotides each, in the highly conserved *B. abortus* AbcR sRNAs. The data demonstrate how the AbcR M1 and M2 motifs are essential for proper regulation of mRNA targets in the pathogenic bacterium *B. abortus* (Fig. 8). Upon transcription of *abcR2* and *abcR1* by VtlR and a yet-to-be-described protein, respectively, the two sRNAs adopt similar secondary structures, exposing two single-stranded RNA motifs, CUCCCA (M1) and GUUCCC (M2). To date, only one mRNA target has been identified as being regulated by the M1 motif alone. In contrast, the majority of transcripts are regulated by M2 or by both M1 and M2. We hypothesize that, upon interaction, the sRNA-mRNA complex is targeted for degradation by an RNase, leading to inhibition of a diverse set of ABC-type transport systems. To our knowledge, this is the first report demonstrating the precise mechanism in *Brucella* by which sRNAs interact with and bind to mRNAs. Most importantly, only the M2 sequence in the AbcR sRNAs is critical for the survival of the brucellae within macrophages and mice, highlighting the importance of relatively short sequence elements in mediating the host-bacterium relationship.

## MATERIALS AND METHODS

### Bacterial strains and growth conditions.

*Brucella abortus* strains were grown on Schaedler agar (BD, Franklin Lakes, NJ) supplemented with 5% defibrinated bovine blood (Quad Five, Ryegate, MT) (SBA) or in brucella broth (BD). For cloning, *Escherichia coli* strains (DH5α) were grown on tryptic soy agar (BD) or in Luria-Bertani broth. When necessary, the medium was supplemented with kanamycin (45 μg/ml) or ampicillin (100 μg/ml). All cultures of and experiments utilizing *Brucella* strains were performed in a biosafety level 3 (BSL-3) facility.

### *Brucella abortus* strain constructions. (i) Wild-type plasmid constructs.

The *B. abortus abcR1*, *abcR2*, and double *abcR1 abcR2* reconstruction and deletion plasmids were previously constructed (15). To construct a *B. abortus bab2_0879* reconstruction plasmid, oligonucleotides were designed to amplify the wild-type locus, plus 1 kb upstream and 1 kb downstream, using *Brucella abortus* 2308 genomic DNA as a template and platinum *Pfx* polymerase (Invitrogen, Carlsbad, CA). A complete list of oligonucleotides can be found in Table S2 in the supplemental material. Following amplification, the fragment was digested with BamHI and PstI, ligated into pNPTS138 (35), and transformed into *E. coli* DH5α. The resulting plasmid, pLS012, was sequenced by the Biocomplexity Institute (BI) at Virginia Tech. All plasmids used in this study can be found in Table S3.

10.1128/mBio.00473-17.3 TABLE S2  Oligonucleotide primers used in this study. Sequences are given in the 5′-to-3′ direction. Underlined nucleotides represent restriction endonuclease recognition sites. Bolded nucleotides represent the T7 RNA polymerase promoter sequence. Download TABLE S2, PDF file, 0.3 MB. Copyright © 2017 Sheehan and Caswell. 2017 Sheehan and Caswell This content is distributed under the terms of the Creative Commons Attribution 4.0 International license.

10.1128/mBio.00473-17.4 TABLE S3  Plasmids used in this study. All plasmid names and a brief description of how the plasmid was constructed are given. Download TABLE S3, PDF file, 0.3 MB. Copyright © 2017 Sheehan and Caswell. 2017 Sheehan and Caswell This content is distributed under the terms of the Creative Commons Attribution 4.0 International license.

### (ii) Gene deletion plasmid constructs and *B. abortus* strains.

The *B. abortus bab2_0612* and *bab2_0879* genes were mutated by an unmarked gene excision strategy as previously described (36). Briefly, 1-kb fragments of the upstream and downstream regions of the genes were amplified by PCR using primers listed in Table S2, genomic *B. abortus* 2308 DNA as a template, and platinum *Pfx* polymerase (Invitrogen). The upstream fragments were digested with the BamHI restriction enzyme, while the downstream fragments were digested with the PstI restriction enzyme. All fragments were phosphorylated with polynucleotide kinase (Monserate Biotechnology Group, San Diego, CA). Fragments were then combined in a single ligation mix with BamHI/PstI-digested pNPTS138 and T4 DNA ligase (Monserate Biotechnology Group). The resulting plasmids, pC^3^041 (Δ*bab2_0612*) and pLS001 (Δ*bab2_0879*) were transformed into *B. abortus* 2308 by electroporation, and a merodiploid clone for each was obtained by selection on SBA plus kanamycin (as previously described in references 14, 16, and 17). Kanamycin-resistant colonies were grown in brucella broth for 6 to 8 h and then plated on plates with SBA plus 10% sucrose. Sucrose-resistant, kanamycin-sensitive colonies were screened by PCR for loss of either *bab2_0612* or *bab2_0879*. The isogenic *bab2_0612* mutant derived from *B. abortus* 2308 was named CC069, while the isogenic *bab2_0879* mutant was named LS006. All plasmids described here can be found in Table S3.

### (iii) Site-directed mutagenesis of motifs in AbcR sRNAs.

For all motif mutants, a protocol for site-directed mutagenesis was followed (QuikChange II XL site-directed mutagenesis kit; Agilent Technologies, Santa Clara, CA) with slight modifications. The *abcR1* and *abcR2* plasmids generated by Caswell et al. (15) (pC^3^032 [RC-*abcR1*] and pC^3^033 [RC-*abcR2*]) were used individually as templates for site-directed mutagenesis. For mutagenesis of the motifs in either *abcR1* or *abcR2*, overlapping oligonucleotides were designed which included either a mutated version of the motif 1 (M1) nucleotide sequence (CUCCCA to UGAUAC) or a mutated version of the motif 2 (M2) nucleotide sequence (GUUCCC to ACGUAU). pC^3^032, for *abcR1* mutagenesis, and pC^3^033, for *abcR2* mutagenesis, were used as templates, and *Pfurious* DNA polymerase (Monserate Biotechnology Group) was utilized in subsequent PCRs. All plasmid products were then subjected to DpnI (Thermo Fisher Scientific, Waltham, MA) digestion to remove any remaining template DNA and were transformed into *E. coli* DH5α cells. The resulting plasmids were named pLS013 (*abcR1-M1*^*mut*^), pLS014 (*abcR1-M2*^*mut*^), pLS015 (*abcR2-M1*^*mut*^), and pLS016 (*abcR2-M2*^*mut*^). For generation of double-motif mutants, either pLS013 (*abcR1-M1*^*mut*^) or pLS015 (*abcR2-M1*^*mut*^) was used as a template, and primers for M2 mutagenesis were utilized. The resulting plasmid constructs were named pLS017 (*abcR1-M1*/*2*^*mut*^) and pLS018 (*abcR2-M1*/*2*^*mut*^).

Motif mutagenesis of *B. abortus abcR1* or *abcR2* was carried out using a *sacB* counterselection strategy, as described above. Plasmids pLS015 (*abcR2-M1*^*mut*^), pLS016 (*abcR2-M2*^*mut*^), and pLS017 (*abcR1-M1*/*2*^*mut*^) were introduced individually into the *B. abortus abcR1 abcR2* double deletion strain (previously generated as described in reference 15 [CC097]) via electroporation. The pNPTS138 backbone plasmid contains a kanamycin resistance marker gene and a *sacB* gene for counterselection on sucrose. Following electroporation, merodiploid clones were obtained by selection on SBA-kanamycin. Single kanamycin-resistant colonies were picked, grown for 6 to 8 h in brucella broth, and plated on SBA containing 10% sucrose. Colony PCR, utilizing confirmation oligonucleotides, was employed with sucrose-resistant, kanamycin-sensitive colonies for reconstruction of either the *abcR1* locus or the *abcR2* locus, and the resulting *B. abortus* strains were named LS030 (*abcR2-M1*^*mut*^), LS038 (*abcR2-M2*^*mut*^), and LS039 (*abcR1-M1*/*2*^*mut*^). For generation of double *abcR1 abcR2* motif mutants, *B. abortus* strains LS030, LS038, and LS039 were subjected to electroporation with pLS013 (*abcR1-M1*^*mut*^), pLS014 (*abcR1-M2*^*mut*^), and pLS018 (*abcR2-M1*/*2*^*mut*^), respectively. Following resolution of sucrose-resistant, kanamycin-sensitive colonies, PCR was employed to identify reconstruction of either the *abcR1* locus or the *abcR2* locus. The resulting *B. abortus* strains were named LS032 (*abcR1*/*2-M1*^*mut*^; referred to here as *abcR-M1*^*mut*^), LS040 (*abcR1*/*2-M2*^*mut*^; referred to here as *abcR-M2*^*mut*^), and LS041 (*abcR1*/*2-M1*/*2*^*mut*^; referred to here as *abcR-M1*/*2*^*mut*^). A schematic of the strains constructed can be found in Fig. 3B.

### Site-directed mutagenesis of M2 in *bab2_0879*.

Similarly to the *abcR* site-directed mutagenesis, plasmid pLS012 (*bab2_0879*) was used as the template and *Pfurious* DNA polymerase was utilized for subsequent PCRs. Following plasmid mutagenesis, DpnI treatment was carried out and the plasmid was transformed into *E. coli* DH5α. For *bab2_0879*, the putative M2 site located in the 5′ UTR was mutated from UAAGGG to UGCAUA. The resulting plasmid was named pLS023 (*bab2_0879-M2*^*mut*^). Construction of a *B. abortus bab2_0879* M2 mutant strain was carried out with the *sacB* counterselection strategy as described above. For plasmid pLS023 (*bab2_0879-M2*^*mut*^), electroporation was performed with *B. abortus* Δ*bab2_0879*. The resulting strain was named LS054 (*bab2_0879-M2*^*mut*^). A schematic of the strains constructed can be found in Fig. 5A.

### Northern blot analysis.

RNA was isolated from *Brucella* strains grown in brucella broth as previously described (36). Briefly, 10 µg of RNA was separated on a denaturing 10% polyacrylamide gel with 7 M urea and 1× TBE (89 mM Tris base, 89 mM boric acid, 2 mM EDTA). For identification of sizes, a low-molecular-weight ladder (New England Biolabs, Ipswich, MA) was radiolabeled with (γ-^32^P)ATP (PerkinElmer, San Jose, CA, USA) and polynucleotide kinase (Monserate Biotechnology Group). Following electrophoresis, the radiolabeled ladder and RNA samples were transferred to an Amersham Hybond-N^+^ membrane (GE Healthcare, Piscataway, NJ) by electroblotting in 1× TBE buffer for 2 h. Following transfer, membranes were subjected to UV cross-linking to the membrane and then prehybridized in ULTRAhyb-Oligo buffer (Thermo Fisher Scientific) for 2 h at ~45°C in a rotating hybridization oven. The oligonucleotide probes (AbcR1/AbcR2 [for detection of both wild-type AbcR1 and wild-type AbcR2], AbcR1-M1^mut^ [for detection of AbcR1 with M1 mutated], AbcR2-M1^mut^ [for detection of AbcR2 with M1 mutated], AbcR1/AbcR2-M2^mut^ [for detection of AbcR1 and AbcR2 with M2 mutated], and 5S-Northern [for detection of 5S ribosomal RNA]) were end labeled with (γ-^32^P)ATP and polynucleotide kinase. The radiolabeled probes were incubated with the prehybridized membranes overnight (~12 h). The following day, all membranes were washed four times for 30 min each time with 2× SSC (1× SSC is 0.15 M NaCl plus 0.015 M sodium citrate), 1× SSC, 0.5× SSC, and 0.025× SSC, each containing 0.1% sodium dodecyl sulfate (SDS), at ~45°C in a rotating hybridizing oven. All membranes were exposed to X-ray film and subsequently visualized by autoradiography.

### Quantitative reverse transcriptase PCR (qRT-PCR).

RNA was isolated from *Brucella abortus* strains as previously described (36). Briefly, *B. abortus* strains were grown to an appropriate phase of growth (late exponential to early stationary phase) in brucella broth with constant shaking at 37°C. For storage, an equal volume of cold 1:1 ethanol-acetone was added to cultures and stored at −80°C for up to 1 month. For RNA isolation, the cell/ethanol-acetone mixtures were thawed for ~10 min at room temperature, and cells were pelleted at 16,000 × *g* for 3 min. RNA was isolated from cells by the use of TRIzol reagent (Invitrogen, Carlsbad, CA) and subsequent ethanol precipitation.

Following RNA isolation, genomic DNA was removed by use of DNase I (Thermo Fisher Scientific, Waltham, MA) as previously described (36). Briefly, 30 µg of RNA was incubated with DNase I (2 U/μl) at 37°C for 1 h. Following incubation, samples were cleaned up by phenol-chloroform extraction and subsequent ethanol precipitation. All RNA samples were resuspended in nuclease-free H_2_O, and their concentrations and purity were checked for by the use of a NanoDrop 1000 spectrophotometer (Thermo Fisher Scientific). All samples had an *A*_260_/*A*_280_ ratio of ~2.0 with a yield of ~1 μg/μl. Following isolation, cDNA was synthesized. For this, ~1 μg of RNA was added to 4 μl of 5× qScript cDNA SuperMix (QuantaBio, Beverly, MA) and brought to a final volume of 20 μl with nuclease-free H_2_O. The qScript cDNA SuperMix contained the following ingredients: optimized concentrations of MgCl_2_, dATP, dCTP, dGTP, and dTTP; recombinant RNase inhibitor protein (RIP); qScript reverse transcriptase; and titrated concentrations of a random hexamer and oligo(dT) primer. The following PCR was carried out for RT of all samples: 5 min at 25°C; 30 min at 42°C; and 5 min at 85°C.

Following RT-PCR, cDNA was utilized for quantitative PCR. As a control for all experiments, 16S rRNA primers were used. cDNA samples were diluted 1:50 in nuclease-free H_2_O, and 2 μl of diluted cDNA was added to 12.5 μl of 2× iTaq Universal SYBR green SuperMix (Bio-Rad, Hercules, CA) and 1.5 μl of 5 μM primer mixture (i.e., forward and reverse), and the reaction mixture was brought to a final volume of 25 μl with nuclease-free H_2_O. The SuperMix contained iTaq DNA polymerase, deoxynucleoside triphosphates (dNTPs), MgCl_2_, SYBR green I dye, enhancers, stabilizers, and a blend of dyes. Gene-specific primers were generated (Table S2) to assess the relative expression levels of *B. abortus* AbcR-specific targets (i.e., *bab1_0313*, *bab1_0314*, *bab1_1794*, *bab1_1799*, *bab2_0491*, *bab2_0612*, *bab2_0879*, and *bab2_1062*). All amplicon lengths were ~150 bp, and all samples were run in triplicate on quantitative PCR (qPCR) plates. qRT-PCR parameters included a single denaturing step for 3 min at 95°C, followed by 40 cycles (denaturing for 15 s at 95°C, annealing for 15 s at 51°C, and extension for 20 s at 72°C) of amplification. Fluorescence representing SYBR green incorporation was analyzed by the use of an iCycler machine (Bio-Rad, Hercules, CA), and the relative levels of mRNA abundance were subsequently assessed using the Pfaffl equation (37).

### Electrophoretic mobility shift assays (EMSAs).

All RNA was generated by *in vitro* transcription with the T7 RNA polymerase promoter and a MAXIscript T7 kit (Ambion, Austin, TX) as previously described (15). Briefly, primers *bab2_0879*-T7-For and *bab2_0879*-T7-Rev were used to amplify 215 nucleotides corresponding to the 5′ UTR and the beginning CDR of the *bab2_0879* mRNA. Similarly, primers *abcR1*-T7-For and *abcR1*-T7-Rev were used to amplify *abcR1* (100 nucleotides), and primers *abcR2*-T7-For and *abcR2*-T7-Rev were used to amplify *abcR2* (110 nucleotides). All fragments were amplified with *Taq* polymerase and cloned into pGEM-T Easy vector (Promega, Madison, WI). Plasmid DNA was purified from putative clones and subjected to sequencing at the BI at Virginia Tech. For control experiments, the previously constructed plasmid with the 5′ UTR of *babR* was utilized (36). Descriptions of all primers and plasmids can be found in Table S2 and Table S3, respectively.

Single-stranded RNA was then generated according to the manufacturer’s instructions. Briefly, the cloned fragments were excised from the pGEM-T Easy backbone by use of the restriction enzymes NcoI and NheI. Following purification of the fragments, *in vitro* transcription was carried out. For generation of radiolabeled probes, BAB2_0879 mRNA and BabR mRNA were uniformly incorporated with (α-^32^P)UTP (PerkinElmer).

For RNA-RNA binding assays, increasing amounts of either AbcR1 or AbcR2 were mixed with either radiolabeled BAB2_0879 mRNA or BabR mRNA. Binding reactions were performed in a 1× structure buffer (Ambion) and tRNA (1 μg/μl) and were brought to a final volume of 20 μl with nuclease-free H_2_O. All binding reactions were heated to 90°C for 2 min and then incubated at room temperature for 30 min. Binding reaction products were then subjected to electrophoresis on a native 6% polyacrylamide gel in 0.5× TBE running buffer on ice for ~1 h at room temperature. Following electrophoresis, gels were dried on 3MM Whatman paper for 1 h at 80°C using a vacuum gel dryer system. Gels were then visualized by autoradiography.

### Virulence of *Brucella* mutant strains in murine macrophages and experimentally infected mice.

To determine the virulence of the *Brucella abcR-M1*^*mut*^, *abcR-M2*^*mut*^, and *abcR-M1*/*2*^*mut*^ strains, experiments using murine peritoneal macrophages were carried out as previously described (38). Murine macrophages were harvested from BALB/c mice and seeded into 96-well plates with Dulbecco’s modified Eagle’s medium (3 wells per *Brucella* strain) supplemented with 5% fetal bovine serum. Macrophages were then infected with opsonized brucellae at a multiplicity of infection (MOI) of 50:1 and were incubated at 37°C for 2 h. Following incubation, infected macrophages were treated with gentamicin (50 μg/ml) for 1 h to eliminate extracellular brucellae. Macrophages were then lysed with 0.1% deoxycholate, and serial dilutions were then plated on SBA to count CFUs. For the 24-h and 48-h time points, macrophages were washed once with PBS following gentamicin treatment, and fresh cell culture medium with gentamicin (20 μg/ml) was added to all macrophages. At the indicated time points, macrophages were lysed with 0.1% deoxycholate, and serial dilutions were plated on SBA in triplicate to count CFUs.

Determination of the ability of *Brucella* mutant strains to infect and colonize BALB/c mice was achieved as previously described (38). Six-week-old female BALB/c mice were infected intraperitoneally with ~5 × 10^4^ CFU of each *Brucella* strain suspended in sterile PBS. Following infection, mice were sacrificed at 1, 4, and 8 weeks postinfection, and spleen homogenates were serially diluted and plated on SBA. For the strains listed in Fig. 6 and 7B, five mice were sacrificed per strain per time point. For the strains listed in Fig. 7A, five mice were sacrificed per strain at the 4-week time point, and four mice were sacrificed per strain at the 8-week time point. All data presented were analyzed utilizing JMP 12.0.0 statistical software (SAS Institute, Cary, NC).

## References

[1] Waters LS, Storz G 2009 Regulatory RNAs in bacteria. Cell 136:615–628. doi:10.1016/j.cell.2009.01.043. 19239884 PMC3132550

[2] Gottesman S, Storz G 2011 Bacterial small RNA regulators: versatile roles and rapidly evolving variations. Cold Spring Harb Perspect Biol 3. doi:10.1101/cshperspect.a003798. PMC3225950 20980440

[3] Møller T, Franch T, Højrup P, Keene DR, Bächinger HP, Brennan RG, Valentin-Hansen P 2002 Hfq: a bacterial Sm-like protein that mediates RNA-RNA interaction. Mol Cell 9:23–30. 11804583 10.1016/s1097-2765(01)00436-1

[4] Sauter C, Basquin J, Suck D 2003 Sm-like proteins in eubacteria: the crystal structure of the Hfq protein from *Escherichia coli*. Nucleic Acids Res 31:4091–4098. doi:10.1093/nar/gkg480. 12853626 PMC167641

[5] Vogel J, Luisi BF 2011 Hfq and its constellation of RNA. Nat Rev Microbiol 9:578–589. doi:10.1038/nrmicro2615. 21760622 PMC4615618

[6] Fröhlich KS, Vogel J 2009 Activation of gene expression by small RNA. Curr Opin Microbiol 12:674–682. doi:10.1016/j.mib.2009.09.009. 19880344

[7] Gottesman S 2011 Roles of mRNA stability, translational regulation, and small RNAs in stress response regulation, p 59–73. *In* Storz G, Hengge R (ed), Bacterial stress responses, 2nd ed. ASM Press, Washington, DC.

[8] De Lay N, Schu DJ, Gottesman S 2013 Bacterial small RNA-based negative regulation: Hfq and its accomplices. J Biol Chem 288:7996–8003. doi:10.1074/jbc.R112.441386. 23362267 PMC3605619

[9] del Val C, Rivas E, Torres-Quesada O, Toro N, Jiménez-Zurdo JI 2007 Identification of differentially expressed small non-coding RNAs in the legume endosymbiont *Sinorhizobium meliloti* by comparative genomics. Mol Microbiol 66:1080–1091. doi:10.1111/j.1365-2958.2007.05978.x. 17971083 PMC2780559

[10] Vercruysse M, Fauvart M, Cloots L, Engelen K, Thijs IM, Marchal K, Michiels J 2010 Genome-wide detection of predicted non-coding RNAs in *Rhizobium etli* expressed during free-living and host-associated growth using a high-resolution tiling array. BMC Genomics 11:53. doi:10.1186/1471-2164-11-53. 20089193 PMC2881028

[11] Wilms I, Voss B, Hess WR, Leichert LI, Narberhaus F 2011 Small RNA-mediated control of the *Agrobacterium tumefaciens* GABA binding protein. Mol Microbiol 80:492–506. doi:10.1111/j.1365-2958.2011.07589.x. 21320185

[12] Torres-Quesada O, Millán V, Nisa-Martínez R, Bardou F, Crespi M, Toro N, Jiménez-Zurdo JI 2013 Independent activity of the homologous small regulatory RNAs AbcR1 and AbcR2 in the legume symbiont *Sinorhizobium meliloti*. PLoS One 8:e68147. doi:10.1371/journal.pone.0068147. 23869210 PMC3712013

[13] Torres-Quesada O, Reinkensmeier J, Schlüter JP, Robledo M, Peregrina A, Giegerich R, Toro N, Becker A, Jiménez-Zurdo JI 2014 Genome-wide profiling of Hfq-binding RNAs uncovers extensive post-transcriptional rewiring of major stress response and symbiotic regulons in *Sinorhizobium meliloti*. RNA Biol 11:563–579. doi:10.4161/rna.28239. 24786641 PMC4152363

[14] Sheehan LM, Budnick JA, Blanchard C, Dunman PM, Caswell CC 2015 A LysR-family transcriptional regulator required for virulence in Brucella abortus is highly conserved among the α-proteobacteria. Mol Microbiol 98:318–328. doi:10.1111/mmi.13123. 26175079 PMC5846693

[15] Caswell CC, Gaines JM, Ciborowski P, Smith D, Borchers CH, Roux CM, Sayood K, Dunman PM, Roop RM II 2012 Identification of two small regulatory RNAs linked to virulence in *Brucella abortus* 2308. Mol Microbiol 85:345–360. doi:10.1111/j.1365-2958.2012.08117.x. 22690807 PMC3391331

[16] Overlöper A, Kraus A, Gurski R, Wright PR, Georg J, Hess WR, Narberhaus F 2014 Two separate modules of the conserved regulatory RNA AbcR1 address multiple target mRNAs in and outside of the translation initiation region. RNA Biol 11:624–640. doi:10.4161/rna.29145. 24921646 PMC4152367

[17] Caswell CC, Oglesby-Sherrouse AG, Murphy ER 2014 Sibling rivalry: related bacterial small RNAs and their redundant and non-redundant roles. Front Cell Infect Microbiol 4:151. doi:10.3389/fcimb.2014.00151. 25389522 PMC4211561

[18] Shah P, Swiatlo E 2008 A multifaceted role for polyamines in bacterial pathogens. Mol Microbiol 68:4–16. doi:10.1111/j.1365-2958.2008.06126.x. 18405343

[19] Khan AU, Di Mascio P, Medeiros MH, Wilson T 1992 Spermine and spermidine protection of plasmid DNA against single-strand breaks induced by singlet oxygen. Proc Natl Acad Sci U S A 89:11428–11430. doi:10.1073/pnas.89.23.11428. 1454831 PMC50564

[20] Ha HC, Sirisoma NS, Kuppusamy P, Zweier JL, Woster PM, Casero RA 1998 The natural polyamine spermine functions directly as a free radical scavenger. Proc Natl Acad Sci U S A 95:11140–11145. doi:10.1073/pnas.95.19.11140. 9736703 PMC21609

[21] Jung IL, Kim IG 2003 Polyamines and glutamate decarboxylase-based acid resistance in *Escherichia coli*. J Biol Chem 278:22846–22852. doi:10.1074/jbc.M212055200. 12670930

[22] Delrue RM, Lestrate P, Tibor A, Letesson JJ, De Bolle X 2004 *Brucella* pathogenesis, genes identified from random large-scale screens. FEMS Microbiol Lett 231:1–12. doi:10.1016/S0378-1097(03)00963-7. 14979322

[23] Becker A, Overlöper A, Schlüter JP, Reinkensmeier J, Robledo M, Giegerich R, Narberhaus F, Evguenieva-Hackenberg E 2014 Riboregulation in plant-associated α-proteobacteria. RNA Biol 11:550–562. doi:10.4161/rna.29625. 25003187 PMC4152362

[24] Mackie GA 2013 RNase E: at the interface of bacterial RNA processing and decay. Nat Rev Microbiol 11:45–57. doi:10.1038/nrmicro2930. 23241849

[25] Valentin-Hansen P, Eriksen M, Udesen C 2004 The bacterial Sm-like protein Hfq: a key player in RNA transactions. Mol Microbiol 51:1525–1533. doi:10.1111/j.1365-2958.2003.03935.x. 15009882

[26] Aiba H 2007 Mechanism of RNA silencing by Hfq-binding small RNAs. Curr Opin Microbiol 10:134–139. doi:10.1016/j.mib.2007.03.010. 17383928

[27] Brennan RG, Link TM 2007 Hfq structure, function and ligand binding. Curr Opin Microbiol 10:125–133. doi:10.1016/j.mib.2007.03.015. 17395525

[28] Ikeda Y, Yagi M, Morita T, Aiba H 2011 Hfq binding at RhlB-recognition region of RNase E is crucial for the rapid degradation of target mRNAs mediated by sRNAs in *Escherichia coli*. Mol Microbiol 79:419–432. doi:10.1111/j.1365-2958.2010.07454.x. 21219461

[29] Davies BW, Walker GC 2008 A highly conserved protein of unknown function is required by *Sinorhizobium meliloti* for symbiosis and environmental stress protection. J Bacteriol 190:1118–1123. doi:10.1128/JB.01521-07. 18055601 PMC2223554

[30] Davies BW, Köhrer C, Jacob AI, Simmons LA, Zhu J, Aleman LM, RajBhandary UL, Walker GC 2010 Role of *Escherichia coli* YbeY, a highly conserved protein, in rRNA processing. Mol Microbiol 78:506–518. doi:10.1111/j.1365-2958.2010.07351.x. 20807199 PMC2959132

[31] Jacob AI, Köhrer C, Davies BW, RajBhandary UL, Walker GC 2013 Conserved bacterial RNase YbeY plays key roles in 70S ribosome quality control and 16S rRNA maturation. Mol Cell 49:427–438. doi:10.1016/j.molcel.2012.11.025. 23273979 PMC3570609

[32] Saramago M, Peregrina A, Robledo M, Matos RG, Hilker R, Serrania J, Becker A, Arraiano CM, Jiménez-Zurdo JI 2017 *Sinorhizobium meliloti* YbeY is an endoribonuclease with unprecedented catalytic features, acting as silencing enzyme in riboregulation. Nucleic Acids Res 45:1371–1391. doi:10.1093/nar/gkw1234. 28180335 PMC5388416

[33] Condon C 2010 What is the role of RNase J in mRNA turnover? RNA Biol 7:316–321. doi:10.4161/rna.7.3.11913. 20458164

[34] Domingues S, Moreira RN, Andrade JM, Dos Santos RF, Bárria C, Viegas SC, Arraiano CM 2015 The role of RNase R in trans-translation and ribosomal quality control. Biochimie 114:113–118. doi:10.1016/j.biochi.2014.12.012. 25542646

[35] Spratt BG, Hedge PJ, te Heesen S, Edelman A, Broome-Smith JK 1986 Kanamycin-resistant vectors that are analogues of plasmids pUC8, pUC9, pEMBL8 and pEMBL9. Gene 41:337–342. doi:10.1016/0378-1119(86)90117-4. 3011607

[36] Caswell CC, Gaines JM, Roop RM II 2012 The RNA chaperone Hfq independently coordinates expression of the VirB type IV secretion system and the LuxR-type regulator BabR in *Brucella abortus* 2308. J Bacteriol 194:3–14. doi:10.1128/JB.05623-11. 22020650 PMC3256608

[37] Pfaffl MW 2001 A new mathematical model for relative quantification in real-time RT-PCR. Nucleic Acids Res 29:e45. doi:10.1093/nar/29.9.e45. 11328886 PMC55695

[38] Gee JM, Valderas MW, Kovach ME, Grippe VK, Robertson GT, Ng WL, Richardson JM, Winkler ME, Roop RM II 2005 The *Brucella abortus* Cu,Zn superoxide dismutase is required for optimal resistance to oxidative killing by murine macrophages and wild-type virulence in experimentally infected mice. Infect Immun 73:2873–2880. doi:10.1128/IAI.73.5.2873-2880.2005. 15845493 PMC1087332

