# Supplementary material for: A 6-Nucleotide Regulatory Motif within the AbcR Small RNAs of Brucella abortus Mediates Host-Pathogen Interactions
Source: mBio. 2017 Jun 6;8(3):e00473-17. doi: 10.1128/mBio.00473-17 (PMC5461406; doi:10.1128/mBio.00473-17)
Supplement: TABLE S1 [file mbo003173341st1.pdf]

Table S1

Brucella gene	Complementary Motif 1 (M1)	Location of M1 from start	Complementary Motif 2 (M2)	Location of M2 from start
bab1_0310	C**AGGGU**	+39	**CAAGGG**	-79
bab1_0313	**GAGGGU**	-8	**CAAGG**C	+74
bab1_0314	**GAGGGU**	0	**CAAGGG**	-41
bab1_0764	**G**C**GGGU**	0	**CAAGG**U	+133
bab1_1794	**GAGGGU**	-8	**CAAGG**U	+396
bab1_1799	**G**U**GGGU**	-8	**CAA**C**GG**	+23
bab2_0491	**GA**A**GGU**	+12	**CAAGGG**	-6
bab2_0612	**GA**C**GGU**	-49	**CAAGGG**	-4
bab2_0670	**GAGGGU**	+98	**CAAGGG**	-1
bab2_0699	**GAGGG**G	-7	**CAA**C**GG**	-12
bab2_0879	**GA**C**GGU**	-31	U**AAGGG**	-5
bab2_1046	**GAGGGU**	-15	**CAAGGG**	-3
bab2_1062	**GAGGG**A	-3	**CAA**A**GG**	0
