# Supplementary material for: A 6-Nucleotide Regulatory Motif within the AbcR Small RNAs of Brucella abortus Mediates Host-Pathogen Interactions
Source: mBio. 2017 Jun 6;8(3):e00473-17. doi: 10.1128/mBio.00473-17 (PMC5461406; doi:10.1128/mBio.00473-17)
Supplement: FIG S1 [file mbo003173341sf1.pdf]

AbcR1/AbcR2 Probe

B. abortus 2308

AbcR1 **CUCCCA**UUGCGCACGGUAAGU**GUUCCC**CUCUGGAGGUUUGCC

AbcR2 **CUCCCA**UUUUGCGCAGGCTGAGU**GUUCCC**CUCUGGAGGUUUGCC

B. abortus abcR-M1^{mut}

AbcR1 **UGAUAC**UUGCGCACGGUAAGU**GUUCCC**CUCUGGAGGUUUGCC

AbcR2 **UGAUAC**UUUUGCGCAGGCTGAGU**GUUCCC**CUCUGGAGGUUUGCC

B. abortus abcR-M2^{mut}

AbcR1 **CUCCCA**UUGCGCACGGUAAGU**ACGUAU**CUCUGGAGGUUUGCC

AbcR2 **CUCCCA**UUUUGCGCAGGCTGAGU**ACGUAU**CUCUGGAGGUUUGCC

B. abortus abcR-M1/2^{mut}

AbcR1 **UGAUAC**UUGCGCACGGUAAGU**ACGUAU**CUCUGGAGGUUUGCC

AbcR2 **UGAUAC**UUUUGCGCAGGCTGAGU**ACGUAU**CUCUGGAGGUUUGCC

AbcR1-M1^{mut} Probe

B. abortus 2308

AbcR1 CGUUGUGCCUC**CUCCCA**UUGCGCACGGUAAGU**GUUCCC**

AbcR2 UGCGUUUCCUC**CUCCCA**UUUUGCGCAGGCTGAGU**GUUCCC**

B. abortus abcR-M1^{mut}

AbcR1 CGUUGUGCCUC**UGAUAC**UUGCGCACGGUAAGU**GUUCCC**

AbcR2 UGCGUUUCCUC**UGAUAC**UUUUGCGCAGGCTGAGU**GUUCCC**

B. abortus abcR-M2^{mut}

AbcR1 CGUUGUGCCUC**CUCCCA**UUGCGCACGGUAAGU**ACGUAU**

AbcR2 UGCGUUUCCUC**CUCCCA**UUUUGCGCAGGCTGAGU**ACGUAU**

B. abortus abcR-M1/2^{mut}

AbcR1 CGUUGUGCCUC**UGAUAC**UUGCGCACGGUAAGU**ACGUAU**

AbcR2 UGCGUUUCCUC**UGAUAC**UUUUGCGCAGGCTGAGU**ACGUAU**

AbcR2-M1^{mut} Probe

***B. abortus* 2308**

AbcR1 CGUUGUGCCUC**CUCCCA**UUGCGCACGGUAAGU**GUUCCC**

AbcR2 UGCGUUUCCUC**CUCCCA**UUUUGCGCAGGCTGAGU**GUUCCC**

B. abortus abcR-M1^{mut}

AbcR1 CGUUGUGCCUC**UGAUAC**UUGCGCACGGUAAGU**GUUCCC**

AbcR2 UGCGUUUCCUC**UGAUAC**UUUUGCGCAGGCTGAGU**GUUCCC**

B. abortus abcR-M2^{mut}

AbcR1 CGUUGUGCCUC**CUCCCA**UUGCGCACGGUAAGU**ACGUAU**

AbcR2 UGCGUUUCCUC**CUCCCA**UUUUGCGCAGGCTGAGU**ACGUAU**

B. abortus abcR-M1/2^{mut}

AbcR1 CGUUGUGCCUC**UGAUAC**UUGCGCACGGUAAGU**ACGUAU**

AbcR2 UGCGUUUCCUC**UGAUAC**UUUUGCGCAGGCTGAGU**ACGUAU**

AbcR-M2^{mut} Probe

***B. abortus* 2308**

AbcR1 **CUCCCA**UUGCGCACGGUAAGU**GUUCCC**CUCUGGAGGUUUGCC

AbcR2 **CUCCCA**UUUUGCGCAGGCTGAGU**GUUCCC**CUCUGGAGGUUUGCC

***B. abortus abcR-M1*^{mut}**

AbcR1 **UGAUAC**UUGCGCACGGUAAGU**GUUCCC**CUCUGGAGGUUUGCC

AbcR2 **UGAUAC**UUUUGCGCAGGCTGAGU**GUUCCC**CUCUGGAGGUUUGCC

***B. abortus abcR-M2*^{mut}**

AbcR1 **CUCCCA**UUGCGCACGGUAAGU**ACGUAU**CUCUGGAGGUUUGCC

AbcR2 **CUCCCA**UUUUGCGCAGGCTGAGU**ACGUAU**CUCUGGAGGUUUGCC

***B. abortus abcR-M1/2*^{mut}**

AbcR1 **UGAUAC**UUGCGCACGGUAAGU**ACGUAU**CUCUGGAGGUUUGCC

AbcR2 **UGAUAC**UUUUGCGCAGGCTGAGU**ACGUAU**CUCUGGAGGUUUGCC
