# Supplementary material for: A 6-Nucleotide Regulatory Motif within the AbcR Small RNAs of Brucella abortus Mediates Host-Pathogen Interactions
Source: mBio. 2017 Jun 6;8(3):e00473-17. doi: 10.1128/mBio.00473-17 (PMC5461406; doi:10.1128/mBio.00473-17)
Supplement: TABLE S2 [file mbo003173341st2.pdf]

Table S2

**Primer Name**	**Sequence (5′-3′)**
bab2_0612-Up-For	GC<u>GGATCC</u>GAGCGCATTGCCCGATCAAA
bab2_0612-Up-Rev	CATGATAGTTCCCTTTATTG
bab2_0612-Down-For	TGATCGCTTGTAAAACTATC
bab2_0612-Down-Rev	GC<u>CTGCAG</u>CATGGGGGCCAGCACATAGC
bab2_0612-Con-For	AACTGCAGTCGATATCTATTGATGCAATAA
bab2_0612-Con-Rev	CAGCAAAGAGTTGATAGTTTTAC
bab2_0879-Up-For	GC<u>GGATCC</u>AAGTTTGAAATGGCGCGAGT
bab2_0879-Up-Rev	CTTCATAGAGCATTCCCCATTTA
bab2_0879-Down-Rev	GC<u>CTGCAG</u>CCCTGATAAACACTGTCCAT
bab2_0879-Down-For	CAATAATTTCCAGTCGCAGCCC
bab2_0879-con-For	GATCACTCCATCTTCCGGAAATC
bab2_0879-con-Rev	TGCCCATGTCAAACTCCAG
bab2_0879-M2-For	TGCCAATAAATGATACGTGCTCTATGAAGT
bab2_0879-M2-Rev	ACTTCATAGAGCACGTATCATTTATTGGCA
bab2_0879-Con-For	GATCACTCCATCTTCCGGAAATC
bab2_0879-Con-Rev	TGCCCATGTCAAACTCCAG
bab2_0879-T7-For	GGTTTGAAATAGATTTCGCGCTGCG
bab2_0879-T7-Rev	GCTA**TAATACGACTCACTATAGGG**ATCCGATCACGTTAGCATGT
abcR1-M1-For	TACCGTGCGCAAGTATCAGAGGCACAACGG
abcR1-M1-Rev	CCGTTGTGCCTCTGATACTTGCGCACGGTA
abcR2-M1-For	CTGCGTTTCCTCTGATACTTTTGCGCAGGC
abcR2-M1-Rev	GCCTGCGCAAAAGTATCAGAGGAAACGCAG
abcR1-M2-For	AAACCTCCAGAGATACGTACTTACCGTGCG
abcR1-M2-Rev	CGCACGGTAAGTACGTATCTCTGGAGGTTT
abcR2-M2-For	AAACCTCCAGAGATACGTACTCAGCCTGCG
abcR2-M2-Rev	CGCAGGCTGAGTACGTATCTCTGGAGGTTT
abcR1-T7-For	GAAAT**TAATACGACTCACTATAGGG**ACTTACCGTTGTGCCTCCTCC
abcR1-T7-Rev	AAAAAAAGAGCCGGACTGGGAA
abcR2-T7-For	GAAAT**TAATACGACTCACTATAGGG**ATCTCAGTGCTGCGTTTC
abcR2-T7-Rev	AAAAAAAAGCCAGACAAACGTCC

AbcR-Northern-WT	GGCAAACCTCCAGAGGGGAACACT
AbcR1-Northern-M1	TGCGCAAGTATCAGAGGCACAACG
AbcR2-Northern-M1	GCGCAAAAGTATCAGAGGAAACGC
AbcR-Northern-M2	GGCAAACCTCCAGAGATACGTACT
5S-Northern	AGTTCGGAATGGGATCGGGTGCAGCC
bab1_0313-RT-For2	ATGGCTGATCTTTCAACGAATTT
bab1_0313-RT-Rev2	TTCCGCGCCGAGCGTTT
bab1_0314-RT-For	TGGCCATCAGCATATCGGTGGCG
bab1_0314-RT-Rev	TTCTTGCCGAAAACATTCTGGGC
bab1_1794-RT-For	GTCTTGTCGTGAATGACGGC
bab1_1794-RT-Rev	GCCTGGCGCGACCAATCCT
bab1_1799-RT-For2	GCTTCTGACGGGTCTGTGC
bab1_1799-RT-Rev2	TTGTTGCGCGGACCCTGC
bab2_0491-RT-For	AGTGCGCATGACGGTTGCG
bab2_0491-RT-Rev	AAATATCGGTGGTCAGCTTTTGC
bab2_0506-RT-For	ATGTGAAACCCAATCCGGTCGAG
bab2_0506-RT-Rev	TCTGTCCGGCCTGAAAG
bab2_0612-RT-For	CAGGGGCTGGATGTCGATATC
bab2_0612-RT-Rev	CCGATAGCGGGGTGGAATAA
bab2_0879-RT-For	TTCGGCGAGCCGGCCTTCACTTT
bab2_0879-RT-Rev	TCGCGTCATGGGGCGGCAACT
bab2_1062-RT-For2	TCCAAGAAAATAAAAACGATGAC
bab2_1062-RT-Rev2	CTTTGCAAAAGGCTGGTAGAATG
16S-RT-For	TCTCACGACACGAGCTGACG
16S-RT-For	CGCAGAACCTTACCAGCCCT
