# Supplementary material for: A 6-Nucleotide Regulatory Motif within the AbcR Small RNAs of Brucella abortus Mediates Host-Pathogen Interactions
Source: mBio. 2017 Jun 6;8(3):e00473-17. doi: 10.1128/mBio.00473-17 (PMC5461406; doi:10.1128/mBio.00473-17)
Supplement: TABLE S3 [file mbo003173341st3.pdf]

Table S3

**Plasmid name**	**Description**	**References or source**
pNPTS138	Cloning vector containing *sacB* gene; Kan^r	35
p*babR*-UTR	5′-UTR of *babR* in pGEM-T Easy	36
pC^{3}032	Wild-type *abcR1* plus 1 kb of upstream and downstream flanking regions in pNPTS138	15
pC^{3}033	Wild-type *abcR2* plus 1 kb of upstream and downstream flanking regions in pNPTS138	15
pC^{3}041	In-frame deletion of *bab2_0612* plus 1 kb of each side flanking region in pNPTS138	This study
pLS001	In-frame deletion of *bab2_0879* plus 1 kb of each side flanking region in pNPTS138	This study
pLS012	Wild-type bab2_0879 plus 1 kb of upstream and downstream flanking regions in pNPTS138	This study
pLS013	*B. abortus abcR1* with mutated M1 sequence (CUCCCA to UGAUAC) in pNPTS138	This study
pLS014	*B. abortus abcR1* with mutated M2 sequence (GUUCCC to ACGUAU) in pNPTS138	This study
pLS015	*B. abortus abcR2* with mutated M1 sequence (CUCCCA to UGAUAC) in pNPTS138	This study
pLS016	*B. abortus abcR2* with mutated M2 sequence (GUUCCC to ACGUAU) in pNPTS138	This study
pLS017	*B. abortus abcR1* with mutated M1 (CUCCCA to UGAUAC) and M2 (GUUCCC to ACGUAU) sequences in pNPTS138	This study
pLS018	*B. abortus abcR2* with mutated M1 (CUCCCA to UGAUAC) and M2 (GUUCCC to ACGUAU) sequences in pNPTS138	This study
pLS023	*B. abortus bab2_0879* with mutated M2 sequence (CAAGGG to UGCAUA) in pNPTS138	This study
pLS024	5′-UTR/beginning of coding region of *bab2_0879* in pGEM-T Easy	This study
